# Supplementary material for: Microfluidic live tracking and transcriptomics of cancer-immune cell doublets link intercellular proximity and gene regulation
Source: Commun Biol. 2022 Nov 12;5:1231. doi: 10.1038/s42003-022-04205-y (PMC9653407; doi:10.1038/s42003-022-04205-y)
Supplement: Supplementary file 2 — Supplementary Information [file 42003_2022_4205_MOESM2_ESM.pdf]

Microfluidic live tracking and transcriptomics of cancer-immune cell doublets link intercellular proximity and gene regulation

Bianca C. T. Flores[1,2,3,9,§], Smriti Chawla[4,§], Ning Ma[1,10,§], Chad Sanada[5,11], Praveen Kumar Kujur[1,12], Rudy Yeung[5,13], Margot B. Bellon[1], Kyle Hukari[5], Brian Fowler[5], Mark Lynch[5,14], Ludmilla T. D. Chinen[2], Naveen Ramalingam[5,*], Debarka Sengupta[4,6,7,8,*], Stefanie S. Jeffrey[1,*].

[1] Department of Surgery, Stanford University School of Medicine, Stanford, CA 94305, USA.

[2] Circulating Tumor Cells Group, A.C.Camargo Cancer Center, São Paulo, SP, 01508-010, Brazil.

[3] Cancer Biology and Epigenetics Group, IPO Porto Research Center (CI-IPOP), Portuguese Oncology Institute of Porto (IPO Porto), 4200-072, Porto, Portugal.

[4] Department for Computational Biology, Indraprastha Institute of Information Technology, New Delhi 110020, India.

[5] New Technologies Group, Fluidigm Corporation, South San Francisco, CA 94080, USA.

[6] Department of Computer Science and Engineering, Indraprastha Institute of Information Technology, New Delhi 110020, India.

[7] Centre for Artificial Intelligence, Indraprastha Institute of Information Technology, New Delhi 110020, India.

[8] Institute of Health and Biomedical Innovation, Queensland University of Technology, Brisbane QLD 4001, Australia.

[9] Current address: Department of Clinical Genetics, Lillebaelt Hospital, Vejle, Denmark.

[10] Current address: Akoya Biosciences, Menlo Park, CA 94025, USA.

[11] Current address: Systems Integration Group, Inscripta Inc, Pleasanton, CA 94588, USA.

[12] Current address: Cancer Biology Laboratory, School of Life Sciences, Jawaharlal Nehru University, New Delhi 110067, India.

[13] Current address: Seer Inc., Redwood City, CA 94065, USA.

[14] Current address: BioSkryb Genomics, Inc., Durham, NC 27713, USA.

[§] These authors contributed equally.

*Correspondence: ssj@stanford.edu (S.S.J.), debarka@iiitd.ac.in (D.S.), naveen.ramalingam@fluidigm.com (N.R.)

Supplementary Figures:

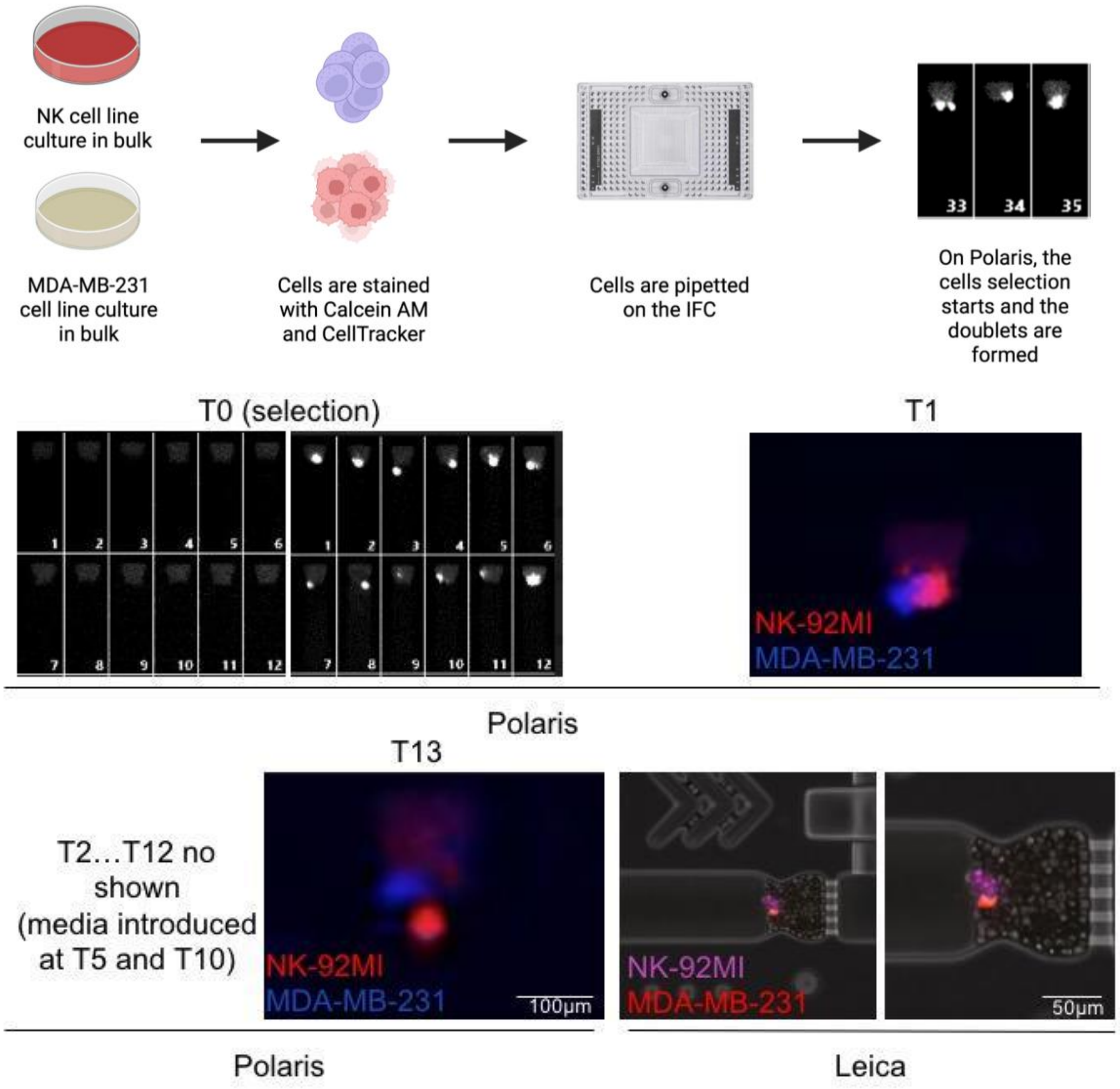

Fig S1. Single-cell or doublets culture and monitoring workflow. The cells were cultured, stained, and pipetted in bulk onto the Polaris IFC. Single cells were selected inside the IFC by the Polaris system using cell viability (Calcien AM) and cell tracker dyes. At the beginning of the experiment (T0 selection step; only singlet wells shown), the NK cells and cancer cells were captured and imaged on the Polaris integrated fluidic circuit (IFC). The representative T0 figure shows the selection step of cell culture chamber 1-12 out of 48 wells on one IFC. After the cell selection, the cells were then incubated and monitored using the Polaris system for 13 hours. Automated images were taken by the Polaris system at a one-hour interval (T0, T1, T2, ...T13). The NK-92MI cells are shown in pseudo red color, and MDA-MB-231 cells are shown in pseudo blue color. These time-lapse images were used for cell-cell distance measurements. The culture media was replenished automatically every five hours on the Polaris system. An image was taken before and immediately after each media change (images T5/T5' and T10/T10'). Before single-cell RNA seq library preparation, high-resolution images were collected for each cell culture chamber using a Leica microscope system for viability tests. The NK-92MI cells are color-coded in pink, and MDA-MB-231 cells are color-coded in red for the Leica microscopic image.

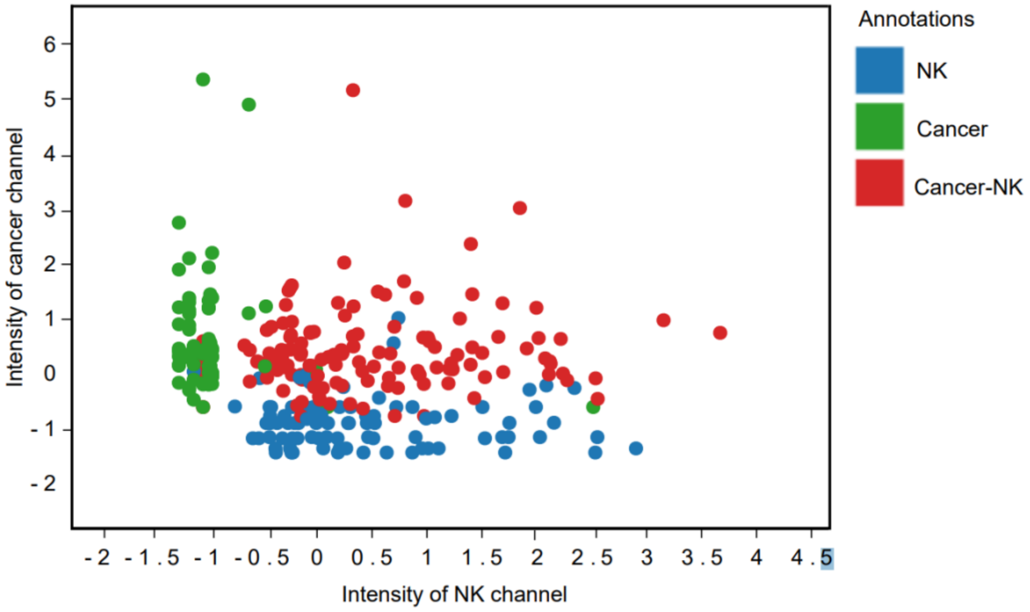

Fig S2. Fluorescence intensities of NK and tumor imaging channels. Scatterplot shows z-score normalized fluorescence intensities of NK and tumor imaging channels, and as per the grouping of cells, these corroborate with the original cell type annotations.

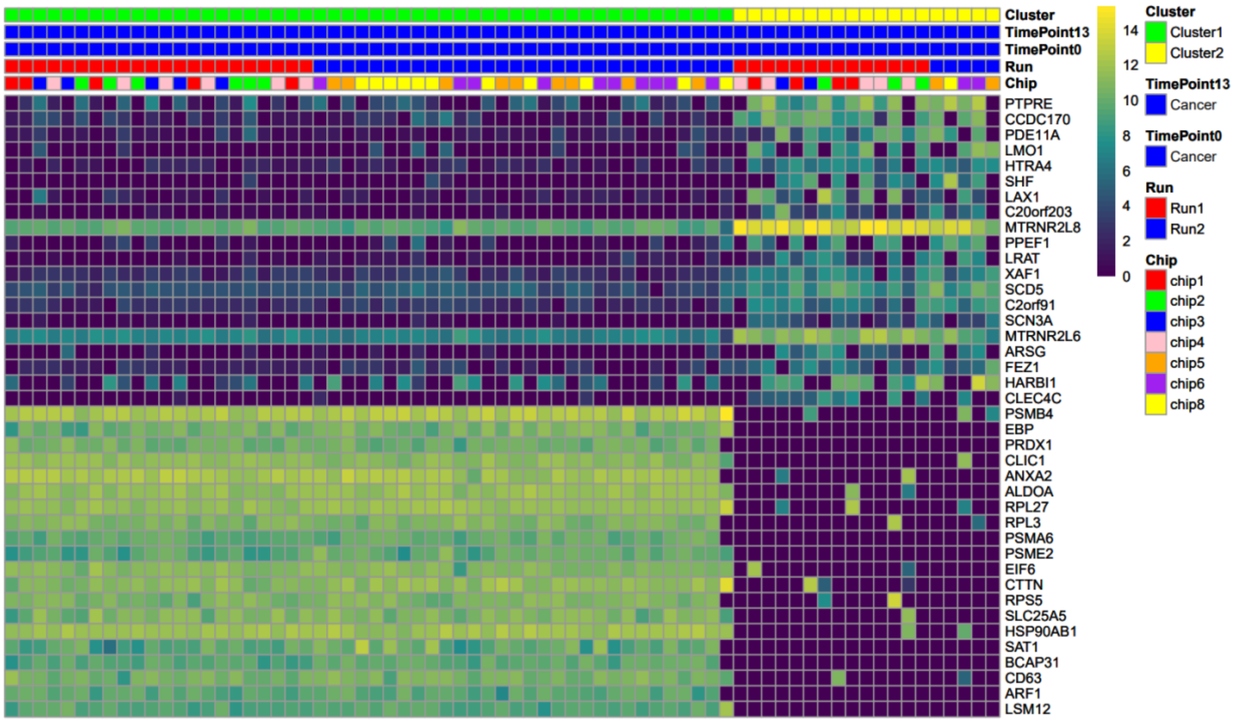

Fig S3. Differentially expressed genes within MDA-MB-231. Heatmap showing differentially expressed genes between two cancer cell line clusters.

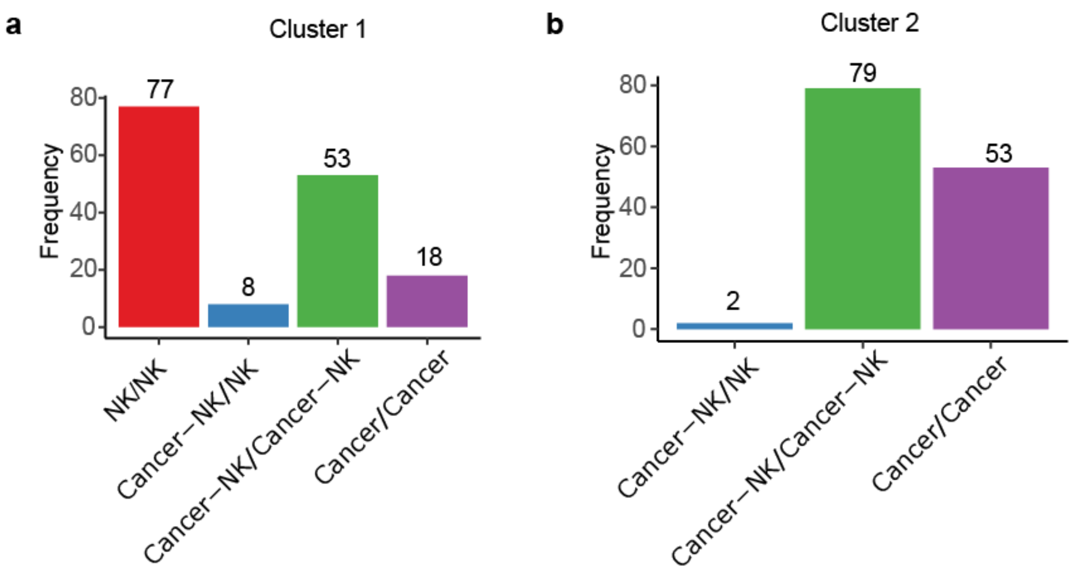

Fig S4. Barplots indicating cell-type frequencies for (a) Cluster 1 and (b) Cluster 2. Legend key indicates cell status at the beginning and the end of the time-course tracking (e.g., CANCER-NK/NK denotes interactions which initially consisted of both Cancer and the NK cells in a chamber, and subsequently the NK cell remained in the chamber after co-incubation due to a cell killing event).
