# Supplementary material for: Microfluidic live tracking and transcriptomics of cancer-immune cell doublets link intercellular proximity and gene regulation
Source: Commun Biol. 2022 Nov 12;5:1231. doi: 10.1038/s42003-022-04205-y (PMC9653407; doi:10.1038/s42003-022-04205-y)
Supplement: Supplementary file 3 — Description of Additional Supplementary Files [file 42003_2022_4205_MOESM3_ESM.pdf]

Description of Additional Supplementary Files

File name: Supplementary Data 1

Description: List of 164 genes associated with Natural killer cell-mediated lysis event of cancer cells and patient survival

File name: Supplementary Data 2

Description: The source data behind the graphs in the paper
