# Supplementary material for: Microfluidic live tracking and transcriptomics of cancer-immune cell doublets link intercellular proximity and gene regulation
Source: Commun Biol. 2022 Nov 12;5:1231. doi: 10.1038/s42003-022-04205-y (PMC9653407; doi:10.1038/s42003-022-04205-y)
Supplement: Supplementary file 6 — Reporting Summary [file 42003_2022_4205_MOESM6_ESM.pdf]

nature portfolio

Corresponding author(s): Dr. Naveen Ramalingam

Last updated by author(s): Oct 25, 2022

Reporting Summary

Nature Portfolio wishes to improve the reproducibility of the work that we publish. This form provides structure for consistency and transparency in reporting. For further information on Nature Portfolio policies, see our Editorial Policies and the Editorial Policy Checklist.

Statistics

For all statistical analyses, confirm that the following items are present in the figure legend, table legend, main text, or Methods section.

n/a	Confirmed	
☐	☒	The exact sample size (n) for each experimental group/condition, given as a discrete number and unit of measurement
☒	☐	A statement on whether measurements were taken from distinct samples or whether the same sample was measured repeatedly
☐	☒	The statistical test(s) used AND whether they are one- or two-sided<br>*Only common tests should be described solely by name; describe more complex techniques in the Methods section.*
☒	☐	A description of all covariates tested
☒	☐	A description of any assumptions or corrections, such as tests of normality and adjustment for multiple comparisons
☒	☐	A full description of the statistical parameters including central tendency (e.g. means) or other basic estimates (e.g. regression coefficient) AND variation (e.g. standard deviation) or associated estimates of uncertainty (e.g. confidence intervals)
☒	☐	For null hypothesis testing, the test statistic (e.g. F, t, r) with confidence intervals, effect sizes, degrees of freedom and P value noted<br>*Give P values as exact values whenever suitable.*
☒	☐	For Bayesian analysis, information on the choice of priors and Markov chain Monte Carlo settings
☒	☐	For hierarchical and complex designs, identification of the appropriate level for tests and full reporting of outcomes
☐	☒	Estimates of effect sizes (e.g. Cohen's d, Pearson's r), indicating how they were calculated

Our web collection on statistics for biologists contains articles on many of the points above.

Software and code

Policy information about availability of computer code

Data collection	*Provide a description of all commercial, open source and custom code used to collect the data in this study, specifying the version used OR state that no software was used.*
Data analysis	R-4.0.0, Seurat v3, PROGgeneV2, RcisTarget(v1.4.0), limma-voom(limma-v3.46.0), ShinyGO(v0.741)

For manuscripts utilizing custom algorithms or software that are central to the research but not yet described in published literature, software must be made available to editors and reviewers. We strongly encourage code deposition in a community repository (e.g. GitHub). See the Nature Portfolio guidelines for submitting code & software for further information.

Data

Policy information about availability of data

All manuscripts must include a data availability statement. This statement should provide the following information, where applicable:

- Accession codes, unique identifiers, or web links for publicly available datasets
- A description of any restrictions on data availability
- For clinical datasets or third party data, please ensure that the statement adheres to our policy

All raw and processed data generated in this study have been submitted to the NCBI Gene Expression Omnibus under accession number GSE181591.

Field-specific reporting

Please select the one below that is the best fit for your research. If you are not sure, read the appropriate sections before making your selection.

☒ Life sciences ☐ Behavioural & social sciences ☐ Ecological, evolutionary & environmental sciences

For a reference copy of the document with all sections, see nature.com/documents/nr-reporting-summary-flat.pdf

Life sciences study design

All studies must disclose on these points even when the disclosure is negative.

Sample size	No sample size calculation was performed.
Data exclusions	For our analysis we have excluded bulk RNA-Seq replicates of each of the NK-92MI and MDA-MB-231 cell-lines from 2 independent runs.
Replication	The single cell gene expression profiles used is this study originated from two independent runs.
Randomization	No randomization was performed in this study.
Blinding	No blinding used in this study.

Reporting for specific materials, systems and methods

We require information from authors about some types of materials, experimental systems and methods used in many studies. Here, indicate whether each material, system or method listed is relevant to your study. If you are not sure if a list item applies to your research, read the appropriate section before selecting a response.

Materials & experimental systems

n/a	Involved in the study	
☐	☒	Antibodies
☐	☒	Eukaryotic cell lines
☒	☐	Palaeontology and archaeology
☒	☐	Animals and other organisms
☒	☐	Human research participants
☒	☐	Clinical data
☒	☐	Dual use research of concern

Methods

n/a	Involved in the study	
☒	☐	ChIP-seq
☒	☐	Flow cytometry
☒	☐	MRI-based neuroimaging

Antibodies

Antibodies used	Alexa Fluor® 647 anti-human CD25 Antibody; Brilliant Violet 785™ anti-human CD69 Antibody; Alexa Fluor® 647 anti-human CD31 Antibody.
Validation	Antibodies were use according to manufacturer's website, and staining was accessed using an SH800S Cell Sorter - Sony Biotechnology.

Eukaryotic cell lines

Policy information about cell lines

Cell line source(s)	ATCC®
Authentication	We identified previously known markers for both NK-92MI cells and MDA-MB-231 cells. In the case of NK cells, we observed strong expression of NK cell marker genes KLRD1, LAIR1, CCR6 and TNFRSF9. Single cancer cells showed TNBC marker genes HMGA1, ANKRD11, and TACSTD2 (Fig. 1B) when analyzed using SCANPY32 toolkit for analyzing single-cell gene expression data.
Mycoplasma contamination	All cell lines were tested negative for mycoplasma contamination.
Commonly misidentified lines (See ICLAC register)	*Name any commonly misidentified cell lines used in the study and provide a rationale for their use.*
